# Effects of Processing Practices (Boiling and Drying) on the Nutritional, Microbial, and Physical Quality Attributes of Small Fish (*Dagaa*) in Small‐Scale Fishery Systems

**DOI:** 10.1002/fsn3.72504

**Published:** 2026-10-19

**Authors:** Patrick Ngwenyama, Patrick Kitabi, Derrick Tunje, Emmanuel Masoud, Pamela Odhiambo, Aditya Parmar, Patrick Kimani

**Affiliations:** ^1^ WorldFish Center Bayan Lepas Penang Malaysia; ^2^ Coastal & Marine Resource Development Mombasa Kenya

**Keywords:** energy efficiency, fish processing, nutrient loss, physicochemical properties, process optimisation, *Rastrineobola argentea*, Sustainable Development Goal 12.3

## Abstract

Small fish *(Dagaa)* are a crucial protein source, but their potential is limited by substantial postharvest losses across the value chain, particularly during processing. Small‐scale fisheries (SSF) rely on salting, boiling, and sun‐drying, yet their effects on quality and energy efficiency remain underexplored. This study evaluated the combined influence of boiling, salting, and drying on the nutritional, microbial, and physical properties of *Dagaa*, and the energy efficiency of *Dagaa* processing. Four processing treatments, namely fresh, salted unboiled‐dried, and salted boiled‐dried under varying boiling times (12 and 15 min), were evaluated. Pre‐boiling in brine significantly increased protein retention and reduced microbial population to levels below permissible regulatory limits relative to unboiled dry salted treatments (*p* < 0.05). Prolonged boiling (15 min vs. 12 min) significantly reduced protein content (*p* < 0.05). Drying increased nutrient concentrations, especially in pre‐boiled salted *Dagaa*. Boiling in a salt solution pretreatment significantly reduced *Dagaa* dimensions, altered morphology, and increased fragmentation and material losses (3%). Unboiled salted sun‐dried *Dagaa* showed lower bulk density, intact morphology, and increased drying surface adhesion. Firewood consumption during boiling averaged 0.17 kg kg^−1^ and 0.20 kg kg^−1^ of *Dagaa*, an estimated annual consumption of 42.5 and 50 t for the 12‐ and 15‐min treatments, respectively, highlighting considerable energy demand and potential environmental implications. A 12‐min salt‐boiling at 85°C–90°C before drying treatment practice achieved the optimum balance of nutrient retention, microbial safety, product quality, drying efficiency, and reduced firewood consumption. These findings inform optimization of marine *Dagaa* processing to maximize product quality, safety, and operational efficiency.

## Graphical abstract

This study found that pre‐boiling in brine enhanced product safety. Prolonged boiling reduced protein content. Boiling pretreatment altered the morphology of dried products. Firewood demand is huge and poses potential long‐term environmental impacts.

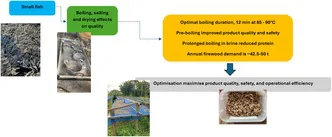

## 1 Introduction

Micronutrient deficiencies remain a major global public health concern, particularly in low‐ and middle‐income regions such as sub‐Saharan Africa (SSA) and Southeast Asia (SEA) (Golden et al. 2021). Often termed “hidden hunger,” these deficiencies persist despite food availability, as predominantly plant‐based diets frequently provide insufficient essential micronutrients, including iron, zinc, selenium, and calcium (Hicks et al. 2019; Robinson et al. 2025). Fish‐based diets can alleviate micronutrient deficiencies in low‐income regions due to their high‐quality protein, omega‐3 fatty acids, vitamins, and bioavailable minerals (Clarke et al. 2022). However, interspecies nutrient variability and limited awareness constrain nutrition‐sensitive fisheries utilization (Kolding et al. 2019; Golden et al. 2021). Small pelagic fish such as *Rastrineobola argentea* (*Dagaa*) are nutrient‐dense and consumed whole, including bones, which enhances micronutrient intake and contributes significantly to the nutritional status of low‐income populations (Kawarazuka and Béné 2011; Ogonda et al. 2014; Kubiriza et al. 2021; Mamun et al. 2025).

Marine *Dagaa* are among the most economically and nutritionally important small pelagic fish species in Kenya's coastal fisheries, especially in Kwale County (Fondo et al. 2025). Recent investments in anchovy processing and export industries further demonstrate the growing economic importance of the sector (Ojijo et al. 2024). In Kenya, the *Dagaa* trade supports more than two million livelihoods and has an estimated annual value of approximately US$200 million (FAO 2026; Wessels et al. 2023).

Although fresh *Dagaa* is preferred by consumers, cooking and drying extend shelf life and enable wider distribution (Oduor‐Odote and Obiero 2009; Katikiro and Mahenge 2022). In low‐income countries, approximately 40% of landings undergo traditional artisanal preservation, mainly smoking, drying, and salting (Robinson et al. 2025). As a result of increased regional trade and cooperation in Africa, processed products from the artisanal sector now constitute a substantial share of intra‐regional trade in small pelagic fish (Kolding et al. 2019).

Fish loss and waste (FLW) is a global challenge, currently estimated at ~35%, prompting SDG 12.3 to call for halving it by 2030 (FAO 2024). *Dagaa* postharvest losses under Small‐Scale Fisheries (SSF) are estimated to range from 20% to 50%, with the highest losses occurring during the rainy season due to a dependence on sun drying and limited cold chain infrastructure (Mgawe 2008). Losses are particularly severe in SSF due to species diversity, perishability, poor infrastructure, and weak policy and market support (Ibengwe 2010; Cederberg and Sonesson 2011; FAO 2024).

*Dagaa* processing is a critical value‐chain activity that reduces postharvest losses and prevents diversion to low‐value uses such as animal feed (Katikiro and Mahenge 2022). Common methods include smoking, sun‐drying, salting, deep frying, fermentation, or combinations thereof, facilitated by whole‐fish processing without prior gutting or cleaning (Abraha et al. 2018; Chaula et al. 2023). While processing increases the shelf life of *Dagaa*, it also results in considerable postharvest losses, both physical and nutritional, yet the effects on nutritional quality are insufficiently documented (Bille and Shemkai 2006; Abelti and Teka 2024).

In low‐income countries, these losses are largely attributable to a reliance on traditional processing methods (FAO 2024). Open sun drying, rather than freezing and cold storage, is the predominant preservation method for *Dagaa* and involves spreading fish on open surfaces (ground or raised platform). However, exposure to direct high temperatures during drying often results in nutrient degradation and compromised product safety due to environmental contamination (Kaponda and Kapute 2018; Kubiriza et al. 2021; Namwanje et al. 2021). Sun drying removes moisture from *Dagaa* through solar radiation, with efficiency dependent on duration and intensity. Slow drying increases the risks of microbial contamination and discoloration (Owaga et al. 2009). Mechanical dryers remain limited, mainly confined to demonstration hubs in low‐income countries (Abdulmajid 2015; Abdul et al. 2024). Boiling fish in brine prior to drying better preserves nutritional quality than boiling in plain water (Birie et al. 2025). However, optimization of boiling temperature and duration is essential, as these parameters can substantially influence the physicochemical, nutritional, sensory, and overall quality of fish products (Fitri et al. 2022). Blanching small fish at approximately 90°C for 5 min may balance microbial and enzyme reduction with quality preservation, although species‐ and process‐specific validation is needed to minimize adverse effects on color, texture, pigments, and nutrient retention (Tran et al. 2022; Fitri et al. 2022; Basak et al. 2023). Smoking is another traditional preservation method in which *Dagaa* is exposed to approximately 70°C in kilns. However, this process generates vapor‐phase polycyclic aromatic hydrocarbons (e.g., pyrene, benzo compounds), promotes protein degradation, poses food safety risks, and reduces lysine bioavailability (Abraha et al. 2018).

The realization of the importance of marine *Dagaa* in improving nutrition in low‐income countries continues to drive research on improving its supply chain. Key postharvest losses of *Dagaa* occur predominantly during processing and transportation, although losses are present at all stages of the value chain (Ward 1996; Mgawe 2008; Akande and Diei‐Ouadi 2010; Assefa et al. 2018; Torell et al. 2020). Nevertheless, assessments at identified hotspots have largely emphasized physical losses, with limited consideration of quality and nutritional losses (Kruijssen et al. 2020).

Although the proximate and micronutrient composition of processed small pelagic fish has been reported, systematic comparative evidence is lacking on how specific cooking practices e.g., boiling time, salting and drying methods, together with their operational parameters (temperature, duration, target moisture), interact to influence nutrient retention and losses in *Dagaa* under smallholder conditions (Ogonda et al. 2014; Clarke et al. 2022). Most existing studies evaluate individual methods in isolation, limiting their usefulness for optimizing integrated processing protocols (Kubiriza et al. 2021).

Current understanding of fish drying kinetics in the coastal Africa region is based mainly on controlled laboratory studies and inadequately represents the variable environmental conditions typical of SSF. As a result, empirical drying curves, moisture diffusivity parameters, and critical moisture thresholds for *Dagaa* under field conditions remain poorly characterized, limiting the effective design and scaling of appropriate drying technologies (Bille and Shemkai 2006; Bellagha et al. 2007; Chanda et al. 2023; Wu et al. 2024).

Boiling is widely used in *Dagaa* processing; however, quantitative data on energy efficiency, fuel consumption, thermal performance and batch capacity of traditional boiling systems are limited (Basak et al. 2023; Birie et al. 2025). Therefore, the current study seeks to (i) assess the influence of various cooking and drying methods and parameters on the physical, nutritional and microbiological composition of *Dagaa*, (ii) determine the characteristics of dried *Dagaa* under smallholder fishery conditions and (iii) investigate *Dagaa* boiling energy efficiency. The study will inform the optimisation of marine *Dagaa* processing techniques to maximize nutritional, product safety, quality and energy/firewood consumption.

## 2 Methods and Materials

### 2.1 Study Site

The study was conducted in August 2025 at Kibuyuni landing site, Kwale County, Mombasa Kenya (−4°07′59.99″ S and 39°12′0.00″ E) (Figure 1). Kibuyuni landing site was purposively selected because it is one of the major marine *Dagaa* production and processing hubs for SSF in Kenya (Aura et al. 2019; Fondo et al. 2025).

**FIGURE 1:**
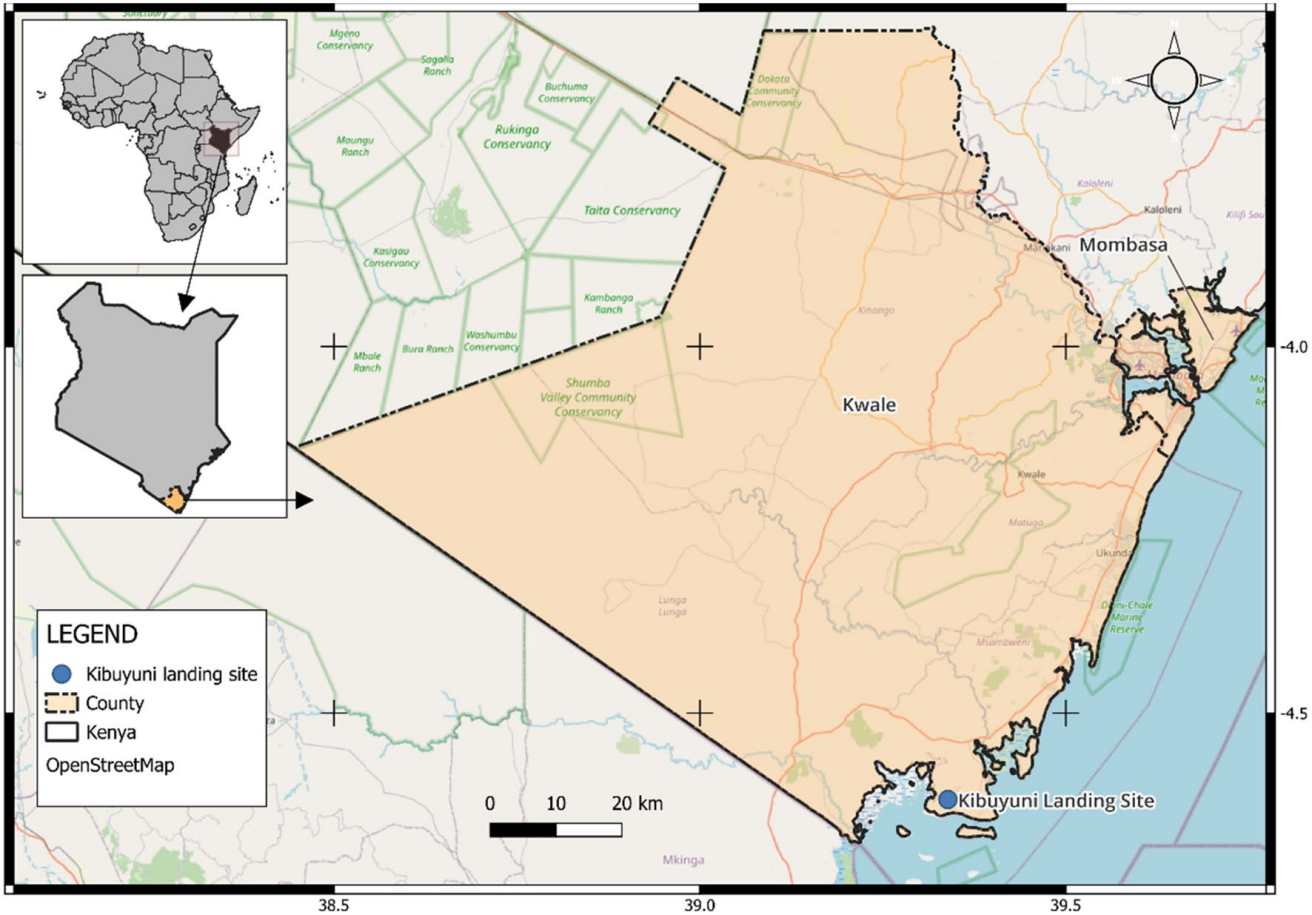
Location of study site—Kiyibuni *Dagaa* landing and processing site, Kenya.

### 2.2 Experimental Design, Trial‐Setting, Sampling, and Sample Analysis

The experiment was conducted using a completely randomized factorial design with replication to evaluate the effects of boiling, boiling duration, and drying on the microbial, nutritional, and physical quality of *Dagaa*. The processing factors were experimentally controlled, and the resulting treatment combinations were randomly assigned to the experimental units. Unboiled treatments were included as reference treatments to enable direct comparison with boiled treatments. Replication was used to estimate experimental variability and improve the precision of treatment comparisons. A completely randomized design was used since no identifiable external nuisance factor requiring control through blocking was present (Balakrishnan et al. 2014).

Four treatments namely: (1) Fresh *Dagaa*, (2) Boiled 12 min salted dried *Dagaa*, (3) Boiled 15 min salted dried *Dagaa*, and (4) Unboiled dry salted dried *Dagaa*, were prepared in triplicate and evaluated for microbial, nutritional (proximate and micronutrients), physical properties (dimensions, bulk density and color), and energy (firewood) consumption. The mean boiling medium temperature was 88°C (Table 1). Each treatment unit was 40 kg fresh *Dagaa*.

**TABLE 1:** The fish processing parameters for the salted boiled and salted unboiled treatments.

	Description	Value
**Salted boiled *Dagaa***		
1	Initial moisture content (%)	54.68 ± 0.55
2	Final moisture content (%)	As per processor practice
3	Amount of boiling water (liters)	140 ± 0.34
4	Heating‐source (fire) temperature (°C)	300 ± 1.05
5	Cooking‐medium temperature (°C)	88 ± 0.76 (85–90)
6	Cooking‐medium salt concentration (g/l)	1.77 ± 0.09
7	Post‐boiling product temperature	79 ± 0.89
8	Dripping time after boiling (min)	5 ± 0.00
9	Cooling time (min)	10 ± 0.00
10	Temperature of product after cooling (°C)	45 ± 0.02
11	Average temperature during drying (°C)	34.38 ± 0.73
12	Average humidity during drying (%)	47.55 ± 0.94
13	Drying time (h)	5 ± 0.00
**Salted unboiled *Dagaa***		
1	Initial moisture content (%)	54.68 ± 0.55
2	Final moisture content (%)	As per processor practice
3	Product temperature before spreading (°C)	26 ± 0.04
4	Amount of dry salt added per 1 kg fresh *Dagaa* (kg)	0.143
5	Average temperature during drying (°C)	32.08 ± 0.44
6	Average humidity during drying (%)	48.30 ± 0.68
7	Drying time (h)	20 ± 0.00

*Note:* Data are presented as mean ± standard error of mean (mean ± SEM, *n* = 3).

For the boiled treatments, fresh *Dagaa* was salted using a brine solution prepared at a concentration of 1.77 g L^−1^ in the cooking medium. For the unboiled treatments, dry salt was applied directly to the fresh fish at a ratio of 1 kg of salt to 7 kg of fresh *Dagaa*, equivalent to approximately 143 g of salt kg^−1^ of fresh fish. The two salting procedures therefore differed in both the mode of salt application and the nominal amount of salt used. These procedures were selected to represent the respective processing practices in Kiyibuni landing site, Kenya. The salted fish were subsequently subjected to the specified drying treatments.

Although salting conditions differed between some processing treatments, this was intentional and reflected the locally practiced methods rather than a laboratory‐standardized salt application. The objective was therefore to evaluate the nutritional and quality outcomes of the processing methods as practiced under local conditions. Consequently, comparisons between boiled and unboiled salted fish represent the combined effects of the respective processing practices, including differences associated with boiling and salt application, rather than the isolated effect of boiling alone.

A limitation of this study is that the salting treatments reflected the common practices used by processors at Kiyibuni; therefore, the salt application method and rate were not standardized on an equivalent basis across treatments. Consequently, the reported salt concentrations were not directly comparable, and treatment effects were interpreted as comparisons of the respective processing practices rather than equivalent salt doses. The present experimental design does not allow the independent effect of boiling to be determined.

#### 2.2.1 Preparation of Treatments

Fresh marine *Dagaa* of known initial weight were placed in perforated aluminium pots and boiled in brine for 12‐ and 15‐min using firewood as the heat source. After boiling, the *Dagaa* were reweighed, cooled, and spread for drying either on raised rack platforms or on ground‐level tarpaulin surfaces. Soon after boiling, the fish were left to drain in perforated boiling containers for approximately 5 min to allow excess brine to drip off, facilitating partial cooling before being spread on the drying platform (Table 1). This step improved handling during transfer and reduced fragmentation of the fish prior to drying. Further cooling for 10 min to a temperature of about 45°C prior to spreading on the drying platform was conducted to minimize fragmentation (Table 1).

HOBO data loggers (Onset Computer Corporation) were placed at the drying platform to continuously record temperature and relative humidity throughout the drying period: 5 and 20 h for salted‐boiled and salted‐unboiled *Dagaa*, respectively. The drying endpoint was determined by processors in accordance with customary practice.

Processing parameters such as post‐boiling product temperature, dripping and cooling time, heating‐source temperature, cooking‐medium temperature, salt concentration, and drying duration were measured and recorded (Table 1). The post‐boiling product temperature was measured in triplicate using an infrared thermometer immediately after boiling. The dripping and cooling period was monitored using a stopwatch in triplicate. The temperature of the heat source (firewood combustion) and the cooking medium were measured in triplicate using an infrared thermometer. Drying duration was recorded using a stopwatch. Salt concentration was determined using the Mohr titration method, as described in Section 2.4.

After determining the drying endpoint, the dried *Dagaa* were winnowed to remove fines and fragments prior to packaging in polypropylene bags for storage and transport. The proportion of fragments and fines was quantified and recorded. The typical boiling and drying equipment is shown in Figure 2.

**FIGURE 2:**
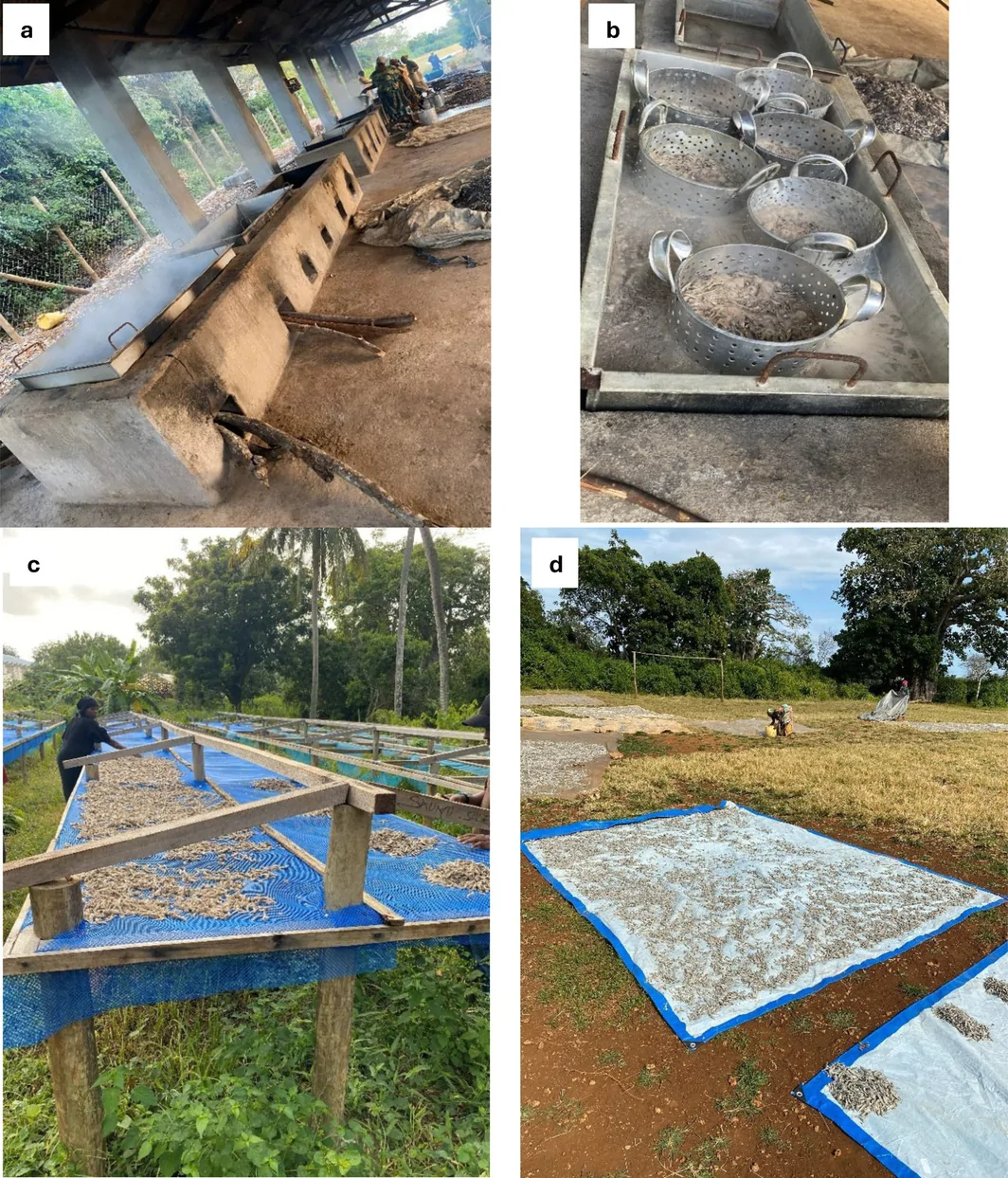
Typical *Dagaa* processing equipment at Kiyibuni landing site: (a, b) boiling units and (c, d) drying facilities.

### 2.3 Determination of the Microbial Load

#### 2.3.1 Preparation of Fish Test Sample

A representative 25 g portion of *Dagaa* from each treatment was minced using a sterile knife. The sample was transferred into a sterile stomacher bag containing 225 mL of sterile 0.1% peptone water and homogenized for 2 min using a stomacher to obtain the initial 10^−1^ suspension (Maturin and Peeler 2001; Birie et al. 2025). The homogenate was thoroughly mixed before aliquots were aseptically withdrawn for serial decimal dilution and inoculation onto the appropriate culture media for enumeration of total aerobic microorganisms, and yeasts and molds. The initial homogenate and subsequent serial dilutions were prepared following the general principles for the preparation of food test samples and initial suspensions described in ISO 6887‐1. Microbial counts were expressed as mean colony‐forming units per gram (cfu/g).

For enumeration, plates containing 15–300 colonies were considered countable for total aerobic plate counts, whereas plates with 10–150 colonies were considered suitable for yeast and mold enumeration. Plates exceeding the respective upper counting limits were recorded as too numerous to count (TNTC), while counts below the method detection limit were reported as below the detection limit. Microbial loads were calculated and expressed as colony‐forming units per gram (cfu/g).

#### 2.3.2 Total Plate Count

Total plate count was enumerated using the pour plate technique in accordance with the ISO 4833:2013 method. Plate count agar was prepared by dissolving agar powder in distilled water and sterilized by autoclaving at 121°C for 15 min, and allowed to cool to about 47°C using a water bath. Serial dilutions (10^−1^ to 10^−7^) of the sample solutions were made. Inoculum (1 ml) from each selected dilution and molten agar were transferred to petri dishes, which were incubated at 37°C for 24 h. Colonies were counted manually using a digital colony counter, and the results were expressed as mean colony‐forming units per gram (cfu/g).

#### 2.3.3 Yeast and Molds

Yeasts and molds were enumerated using the surface spread technique in accordance with ISO 21527‐2:2009. Dichloran Rose Bengal Chloramphenicol (DRBC) agar was weighed and mixed with distilled water. This mixture was sterilized by autoclaving at 121°C for 15 min and immediately cooled to about 47°C. Molten agar was aseptically poured into the petri dish and allowed to set. Inoculum (0.1 ml) from the serial dilutions (10^−1^ to 10^−7^) of the *Dagaa* fish samples was aseptically transferred onto the center of the solidified agar and evenly spread over the agar surface using a sterile wire loop. This set‐up was incubated at room temperature for 5 days. Colonies were counted manually using a digital colony counter, and the results were expressed as mean cfu/g.

### 2.4 Determination of Salt Concentration of Cooking Medium

The salt concentration was measured based on the Mohr argentometric titration method for chloride determination as described in ISO 9297 and standard analytical methods for argentometric titration (Birie et al. 2025; Tao et al. 2025). The brine sample was thoroughly mixed, and a 1 ml aliquot was accurately transferred into a 100 mL volumetric flask and diluted to volume with distilled water. A 10 mL aliquot of the diluted brine was then transferred into a 250 mL Erlenmeyer flask, followed by the addition of approximately 50 mL of distilled water and 1 mL of 5% (w/v) potassium chromate (K_2_CrO_4_) indicator. The solution was titrated with a standardized 0.1000 mol L^−1^ silver nitrate (AgNO_3_) solution until the first persistent brick‐red color was observed, indicating the formation of silver chromate (Ag_2_CrO_4_). A reagent blank was analyzed using the same procedure, and the blank titer was subtracted from the sample titer. All determinations were performed in triplicate. The chloride concentration was calculated from the corrected volume of AgNO_3_ consumed, based on the 1:1 stoichiometric reaction between Ag^+^ and Cl^−^. The salt concentration was expressed as a percentage sodium chloride (NaCl) in the original brine sample using Equation (1).
(1)

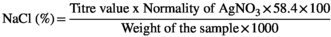




### 2.5 Nutritional Analysis

#### 2.5.1 Preparation of Ground Fish Samples

Analysis was conducted at the Kenya Marine and Fisheries Research Institute (KMFRI) laboratory in Mombasa, Kenya. Representative minced fish sample types were prepared for proximate and micronutrient analyses. Individual treatment samples were homogenized, ground, and blended using a FRITSCH Pulverisette 14 (Germany) to particles small enough to pass through a 1.0 mm sieve, divided into 200 g portions, packaged, and stored at −20°C. Upon usage, batches of individual *Dagaa* treatment representative samples were thawed in a cold room (4°C) for 16 h before analysis.

#### 2.5.2 Proximate Analysis

##### 2.5.2.1 Total Moisture

Total moisture content was determined using the air‐oven gravimetric method following the AOAC Method 950.46 (AOAC 2023). Clean, dry crucibles were oven‐dried (Memmert, Germany), cooled in a desiccator to room temperature, and weighed to constant mass. Approximately 5 g of homogenized fish sample was accurately weighed into each crucible in triplicate and dried at 105°C ± 2°C until constant weight. The dried samples were cooled in a desiccator and reweighed. Moisture content (%) was calculated from the loss in weight as shown in Equation (2).

(2) $$\text{Moisture}\;\left(\%\right)=\frac{w2-w3}{w2-w1}\times 100$$

where: *w*1 = weight of the empty moisture dish (g); *w*2 = weight of the dish plus sample before drying (g); *w*3 = weight of the dish plus dried sample after oven drying (g).

##### 2.5.2.2 Total Ash

Total ash was determined according to the AOAC Method 920.153 (AOAC 2023). Approximately 2.0 g of pre‐dried, homogenized *Dagaa* was weighed into pre‐weighed porcelain crucibles and incinerated at 550°C ± 25°C for 6 h in a muffle furnace CARBOLITE Furnace RHF 1600 (Germany). Crucibles were cooled in a desiccator to constant weight before weighing. Ash content (%) was calculated gravimetrically on a dry‐weight basis (Equation 3).

(3) $$\mathrm{Ash}\;\left(\%\right)=\frac{\text{Weight of}\;\mathrm{ash}}{\text{Original}\;\mathrm{wet}\;\text{weight}}\times 100$$

##### 2.5.2.3 Total Protein

Total protein content was determined using the micro‐Kjeldahl method following the latest AOAC Method 981.10 for nitrogen determination in foods (AOAC 2023). Approximately 0.5 g of homogenized sample was digested with concentrated sulfuric acid (95%–98%, Merck, Germany, 2024) in the presence of Kjeldahl catalyst tablets (K_2_SO^4^/CuSO_4_/TiO_2_; Merck, 2024) using a Kjedahl Digester K‐449 (BÜCHI Labortechnik AG, Switzerland, 2023). The digest was distilled with 40% NaOH using a KjelMaster K‐375 (BÜCHI, 2023), and the liberated ammonia was collected in 4% boric acid and titrated with standardized 0.1 N HCl (Merck, 2024). Nitrogen (%) was converted to crude protein using a factor of 6.25 (Equation 4).

(4) $$\text{Total protein}\;\left(\%\right)=\text{Nitrogen}\;\left(\%\right)\times 6.25$$

##### 2.5.2.4 Total Fat

Total fat content was determined using the Soxhlet solvent extraction method following the principles of the latest AOAC Method 991.36 (AOAC 2023), with lipid recovery enhanced using the Bligh and Dyer (1959) chloroform–methanol extraction procedure. Approximately 2.0 g of homogenized sample was extracted using analytical‐grade petroleum ether (40°C–60°C; Merck, Germany, 2023) in a Soxhlet extraction system (BÜCHI Extraction System B‐811, BÜCHI Labortechnik AG, Flawil, Switzerland, 2023). The chloroform extract was evaporated under reduced pressure, and total lipid content was calculated as indicated in Equation (5).

(5) $$\text{Lipid}\;\left(\%\right)=\frac{\text{lipid mass}\times \text{total chloroform volume}}{\text{sample mass}\times \text{chloroform volume evaporated}}\times 100$$

##### 2.5.2.5 Dietary Fiber

Dietary fiber was determined using the enzymatic‐gravimetric method according to AOAC Method 991.43 (AOAC 2023). Approximately 1.0 g of homogenized sample was sequentially digested with heat‐stable α‐amylase, protease, and amyloglucosidase (Megazyme Ltd., Bray, Ireland) to remove digestible starch and protein. Soluble fiber was precipitated with 95% ethanol (Merck, Germany) and the residue was filtered using a Fibertec 8000 system (FOSS Analytical A/S, Hillerød, Denmark), dried to constant weight, corrected for ash and protein, and expressed as total dietary fiber on a dry‐weight basis following AOAC (2023) procedures.

#### 2.5.3 Micronutrient Analysis

Analysis of iron, calcium, zinc, and magnesium was conducted using the iCAP 6000 Series Inductively Coupled Argon Plasma Mass Spectrometry (ICP‐MS Germany). The sample for analysis was prepared according to the methods of AOAC (2023), and each analysis was carried out in triplicate. All surface areas, materials, equipment and apparatus involved in sample preparation and analysis were kept dust‐free to minimize chance of contamination, since trace amounts of analyte metals are always present in the environment and may be present in dust particles. Fish samples were thoroughly blended to ensure homogeneity prior to removal of a test subsample.

The fish samples were homogenized by grinding to a small particle size to pass through a sieve size of 1.0 mm diameter using FRITSCH Pulverisette 14 (Germany), then dry ashed and digested as described in the AOAC. (2023). A 5 g ground sample was weighed in a crucible, and 1 mL 50% magnesium nitrate was added ensuring that it was well mixed with the sample before it was evaporated on a steam bath. The mixed samples in crucibles were placed in a muffle furnace CARBOLITE Furnace RHF 1600 (Germany) set at 550°C temperature to remove all the carbon until white ash was observed. The crucibles were then removed from the furnace after cooling, and 2 mL of nitric acid was carefully added. The mixture was then transferred to 100 mL volumetric flask and made up to volume. A blank solution was prepared by taking the same amount of acid and following the same steps but using water instead of the test sample. The diluted sample digest was then analyzed using inductively coupled plasma mass spectrometry (ICP‐MS) (APA et al. 2012; AOAC 2023). The metal concentration (mg/100 g) was calculated using Equation (6).

(6) $$\text{Metal concentration}=\frac{\text{Sample reading}-\text{Blank reading}}{\text{Sample weight}}\times 10$$

The basis of the technique was the measurement of masses of charged ions in ICP‐MS. Samples were nebulised and the resulting aerosol was transported into the plasma torch where ionization occurs, and subsequent emission of spectra was produced by the radio frequency inductively coupled plasma (ICP).

### 2.6 Physical Characterization of Processed *Dagaa* (Dimensions, Appearance, and Bulk Density)

Physical quality attributes of *Dagaa* including length and width (dimensional measurements) and appearance (color, shape, integrity of body parts, and ease of removal from the drying platform) were assessed in triplicate and systematically recorded. Sensory evaluation of the dried *Dagaa* was conducted jointly by Kibuyuni processors and researchers.

A qualitative sensory appraisal of the dried fish products was conducted under field conditions using an untrained panel comprising fishers, traders, researchers, and processors with practical experience in the fish value chain. Participants provided open‐ended observations and comments on the color, shape, integrity of body parts, and ease of removing the dried fish from the drying rack. No predefined numerical sensory scores were assigned, and the assessment was not conducted as an independent rating exercise. Instead, participants' comments were recorded and subsequently coded and grouped into recurring themes corresponding to the evaluated product characteristics. The thematic results were used to describe stakeholder perceptions of appearance, physical integrity, and handling characteristics of the dried products.

Bulk density (M/V) of marine *Dagaa* was determined using a volumetric method. Three containers of known volume were used. Each container was filled with fresh *Dagaa* to level (not heaped) and the fish were weighed to obtain the initial mass. The *Dagaa* were then processed according to the four treatments described in Section 2.2. The dried *Dagaa* were placed into the same container, and the weight and height occupied by the dried product were measured. The corresponding volume occupied by the dried *Dagaa* was calculated, and bulk density as the ratio of fish mass (kg) to the volume occupied (m^3^). All measurements and calculations were conducted in triplicate.

### 2.7 Firewood Consumption Evaluation

The quantity of firewood required per kg of fresh *Dagaa* was determined by ratios using the information obtained from Kiyibuni landing site processors, namely: total weight processed, time required, quantity processed per unit time, total amount of firewood used, and cost of the firewood as in Equation (7). The annual estimated total quantity of firewood used by Kiyibuni landing site SSF was calculated by multiplying by the total *Dagaa* production of the area, as given in Equation (8).

(7) $$\mathrm{QF}=\left(\mathrm{TQF}/\mathrm{TPD}\right)$$

where QF = Quantity of firewood to process 1 kg of *Dagaa*, TPD = Total *Dagaa* processed, TQF = Total quantity of firewood used.

(8) $$\mathrm{AFC}=\left(\mathrm{QF}*\mathrm{AFP}\right)$$

where AFC = Annual firewood consumption in Kiyibuni landing site for *Dagaa* processing, AFP = Annual fish processed in Kiyibuni landing site.

The annual cost of firewood is calculated by multiplying the unit price of firewood by the annual estimated total quantity of firewood used in Kiyibuni landing site for *Dagaa* processing.

### 2.8 Data Analysis

Qualitative data from the physical attributes' evaluation were analyzed through systematic summarization of responses across panelists and thematic grouping of attributes. Data on nutritional composition (proximate and micronutrients), *Dagaa* dimensions, and bulk density were entered, coded, and cleaned using Microsoft Excel. Treatment effects on microbial, nutritional quality, physical dimensions, and bulk density were evaluated using analysis of variance (ANOVA) in GenStat (18th edition), following verification of ANOVA assumptions. Where significant differences were observed (*p* < 0.05), treatment means were separated using Tukey's test at the 95% confidence level.

## 3 Results

### 3.1 Microbial Level of Dry‐Salted *Dagaa*

Pre‐boiling in brine prior to drying significantly reduced total plate count (TPC), with values decreasing to 3.2 and 3.0 cfu/g after 12‐ and 15‐min boiling, respectively (*p* < 0.05), well below the maximum permissible limit of 5 cfu/g. In contrast, unboiled dry salted *Dagaa* showed a lower TPC than baseline (6.4 cfu/g) but remained above the regulatory limit and significantly higher than pre‐boiled treatments (Table 2). Yeast and mold counts decreased significantly from 4.5 cfu/g in the fresh *Dagaa* treatment to undetectable levels (0 cfu/g) in both the boiled salted dried and unboiled‐salted dried *Dagaa* treatments (*p* < 0.05) (Table 2).

**TABLE 2:** Microbial level of dry‐salted *Dagaa*.

	Treatment	Total plate count (cfu/g)	Yeast and molds (cfu/g)	Maximum limit (cfu/g)
1	Fresh (baseline)	10.4 ± 0.89^c^	4.5 ± 0.56a	5
2	Boiled 12 min salted dried *Dagaa*	3.2 ± 0.34^a^	0.00 ± 0.00b (undetectable)	
3	Boiled 15 min salted dried *Dagaa*	3.0 ± 0.25^a^	0.00 ± 0.00b (undetectable)	
4	Unboiled salted dried *Dagaa*	6.4 ± 0.72^b^	0.00 ± 0.00b (undetectable)	
	*p*	< 0.05		

*Note:* Data is presented as mean ± standard error of mean (mean ± SEM, *n* = 3). Different superscript letters represent significant differences at 5% probability.

### 3.2 Variation in Proximate and Micronutrient Profiles Among the Dried *Dagaa* Fish Treatments

#### 3.2.1 Moisture Content

The unboiled dry‐salted *Dagaa* treatments exhibited significantly higher moisture levels than the boiled brine‐salted treatments, with mean values of 16.91% and 14.04%, respectively (*p* < 0.05) (Figure 3). Relative to fresh *Dagaa*, moisture content decreased by around 72.5% in the boiled‐salted dried treatments and by 69% in the unboiled‐salted dried treatments. Boiling in brine markedly shortened the drying duration, achieving the target moisture content in 5 h compared with 20 h for the unboiled dry‐salted treatments (Figure 3).

**FIGURE 3:**
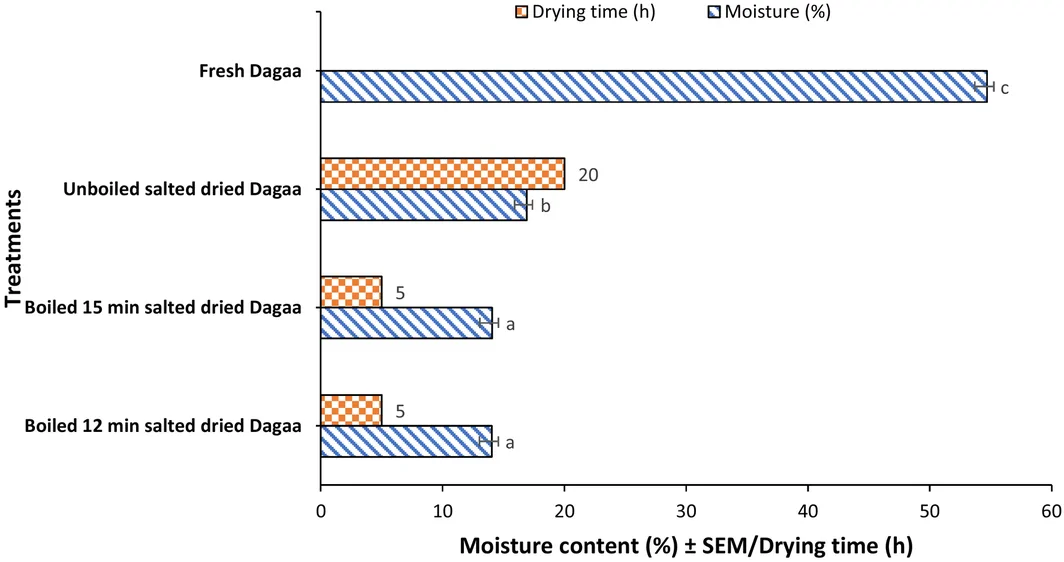
Moisture content of different dried *Dagaa* treatments and drying time. The error bars represent the standard error of the mean (SEM) of triplicate measurements (mean ± SEM, *n* = 3). Different letters represent significant differences at 5% probability.

#### 3.2.2 Proximate Composition

Fresh *Dagaa* contained mean ash, protein, fat, and fiber levels of 5.21%, 20.68%, 5.43%, and 2.23%, respectively (Figure 4). Ash content varied across treatments and increased significantly following drying, with the greatest rise observed in the brine‐salted boiled (12 min) dried *Dagaa* (*p* < 0.05). Protein content also increased significantly after drying (*p* < 0.05), with the highest increase recorded in the salted‐boiled (12 min) dried *Dagaa* and the lowest in the unboiled dry salted dried *Dagaa*. Extended boiling in brine solution (15 min vs. 12 min) significantly reduced the protein content of dried *Dagaa* by approximately 11% (*p* < 0.05). The dry‐salted unboiled dried *Dagaa* exhibited protein contents that were approximately 20% lower than their salted‐boiled counterparts (*p* < 0.05).

**FIGURE 4:**
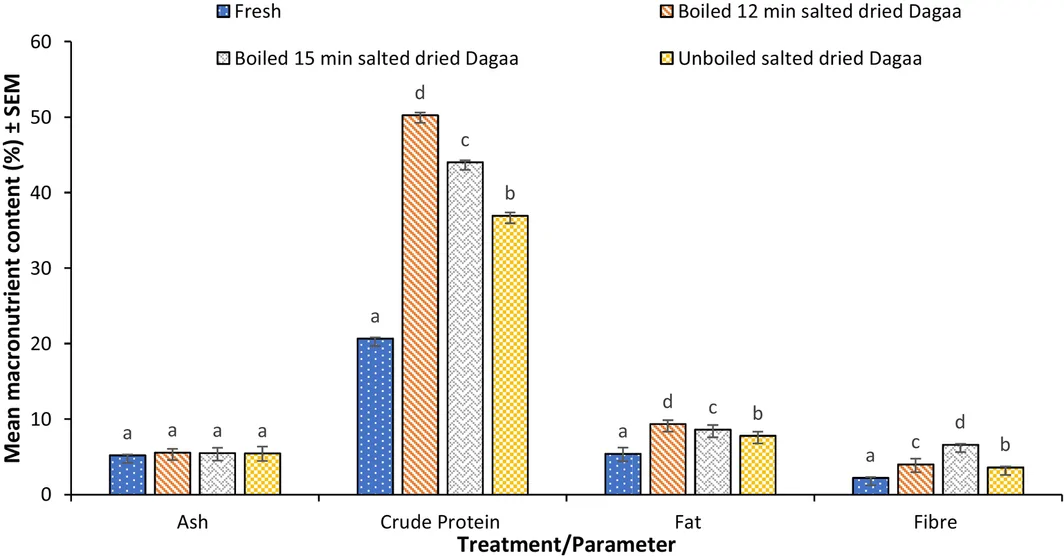
Proximate content of different dried *Dagaa* treatments. The error bars represent the standard error of the mean (SEM) of triplicate measurements (mean ± SEM, *n* = 3). Different letters represent significant differences at 5% probability.

Processing via boiling, salting, and drying led to a marked increase in fat content, with the highest increase observed in *Dagaa* boiled in brine for 12 min (*p* < 0.05). Similarly, processing resulted in a significant increase in fiber content (*p* < 0.05), with the greatest increase in the treatment boiled in brine for 15 min (Figure 4).

#### 3.2.3 Micronutrients

The fresh *Dagaa* contained 12.40, 8.53, 249.70, and 129.70 mg/g of iron, zinc, calcium and magnesium, respectively. The iron, zinc, calcium, and magnesium content increased significantly by approximately 59%, 79%, 66%, and 64%, respectively after processing (salting, boiling, and drying) (*p* < 0.05). The boiled 12 min salted‐dried *Dagaa* treatment had significantly higher levels of iron, zinc, calcium and magnesium than both the boiled (15 min) salted‐dried *Dagaa* and unboiled dry‐salted dried *Dagaa* treatments (*p* < 0.05). Fresh *Dagaa* had a relatively higher level of calcium and magnesium compared to other micronutrients (*p* < 0.05) (Figure 5).

**FIGURE 5:**
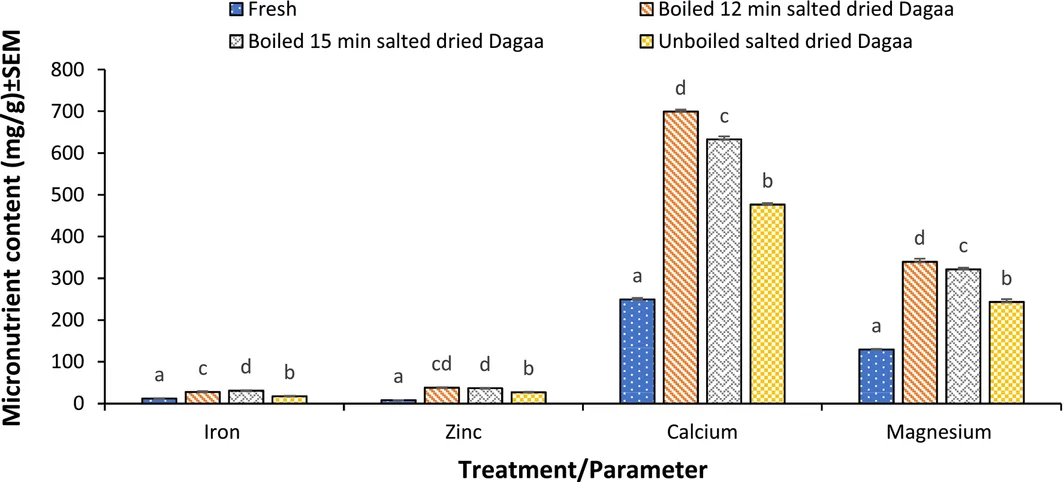
Micronutrient content of different dried *Dagaa* treatments. The error bars represent the standard error of the mean (SEM) of triplicate measurements (mean ± SEM, *n* = 3). Different letters represent significant differences at 5% probability.

### 3.3 Dried *Dagaa* Characteristics (Length, Appearance, and Bulk Density)

Fresh *Dagaa* exhibited a shiny silver color with straight, intact body parts. In contrast, the salted‐boiled sun‐dried treatment showed a golden to dark brown color, bent morphology, and a loss of head and tail, but it was easily detached from the drying rack or tarpaulin (Table 3). The dry‐salted, unboiled, sun‐dried *Dagaa* displayed a pale brown to grayish color and retained straight, intact body parts, but it adhered to the drying surface and to adjacent fish (Table 3).

**TABLE 3:** Physical characteristics of processed *Dagaa* **—**Bulk density, length and width, fragmentation (%) and appearance.

	Treatment	Parameters				
		Length (mm)	Width (mm)	Bulk density (kg/m^3^)	Fragments generated during winnowing (%)	Physical quality*
1	Fresh *Dagaa*	6.41 ± 0.37^a^	0.94 ± 0.10^a^	1075.00 ± 0.05^c^	na	Shiny silver, straight and full body parts
2	Boiled 12 min salted dried *Dagaa*	3.92 ± 0.34^b^	0.37 ± 0.06^b^	357.50 ± 0.12^b^	2.5	Golden dark brown, bent, no head and tail, easy to remove from drying rack
3	Boiled 15 min salted dried *Dagaa*	3.90 ± 0.25^b^	0.34 ± 0.06^b^	351.50 ± 0.18^b^	3	idem
4	Unboiled salted dried *Dagaa*	6.57 ± 0.47^a^	0.83 ± 0.16^a^	217.50 ± 0.08^a^	1	Pale brown to grayish, straight, full body parts, sticky on drying rack
	*p*	*p* < 0.05	*p* < 0.05	*p* < 0.05	na	na

*Note:* Data is presented as mean ± standard error of mean (mean ± SEM, *n* = 3). Different superscript letters represent significant differences at 5% probability.

Abbreviations: idem, the same as previously stated; na, not applicable.

* Color, shape, body parts, removability from the drying rack.

The length and width of *Dagaa* decreased significantly following salting, boiling, and sun drying (*p* < 0.05). However, no significant differences in length or width were observed between fresh *Dagaa* and dry salted‐unboiled sun‐dried *Dagaa* (*p* > 0.05) (Table 3).

Bulk density decreased significantly following drying (*p* < 0.05) (Table 3), with the salted unboiled sun‐dried *Dagaa* exhibiting a significantly lower bulk density than the salted‐boiled sun‐dried *Dagaa* (*p* < 0.05).

After winnowing prior to packaging, *Dagaa* fragments/meal constituted 2.5% and 3.0% of the salted‐boiled sun‐dried treatments processed for 12 and 15 min, respectively, and 1.0% of the dry‐salted, unboiled, sun‐dried treatment (Table 3).

### 3.4 *Dagaa* Boiling Energy Efficiency

Firewood was the source of energy for boiling *Dagaa* at the Kiyibuni landing site. Approximately 0.17 and 0.20 kg of firewood were required to boil 1 kg of fresh *Dagaa* adequately before drying for 12‐ and 15‐min boiling, respectively (Table 4). The annual firewood requirement for processors at the Kiyibuni landing site was estimated at 42.5 t for the 12‐min boiling treatment and 50 t for the 15‐min boiling treatment, translating to costs of USD 2125 and USD 2500, respectively (Table 4).

**TABLE 4:** Quantity and amount of firewood required in boiling *Dagaa*.

	Treatment	Quantity of *Dagaa* (kg)	Quantity of firewood required (kg)	Unit cost (USD)	Total cost (USD)
1	Boiled 12 min salted dried *Dagaa*	1	0.17	0.05	0.008
		250,000*	42,500	0.05	2125.00
2	Boiled 15 min salted dried *Dagaa*	1	0.20	0.05	0.01
		250,000*	50,000	0.05	2500.00

* Estimated total *Dagaa* processed in Kiyibuni landing site per year (Shimoni District Council Statistics, 2024).

## 4 Discussion

### 4.1 Effects of Boiling and Drying *Dagaa* on Product Safety and Nutritional Composition

#### 4.1.1 Microbial Quality

Treatments involving salting combined with boiling consistently maintained TPC within acceptable safety limits (EAS 2014), whereas fresh *Dagaa* deteriorated rapidly under tropical ambient conditions. Boiling, salting, and drying reduced yeast and mold counts in fresh *Dagaa* to undetectable levels, demonstrating the strong antimicrobial effects of combined heat treatment, reduced water activity, and increased salt concentration (Birie et al. 2025). These hurdles inhibit fungal growth and improve microbiological safety and shelf stability of dried fish products (Kigozi et al. 2020; Suprakash et al. 2020). The differences in salting method and salt concentration contributed to variations in microbial quality of the processing practices. The lower microbial counts observed in brine‐salted boiled dried *Dagaa* compared with dry‐salted dried *Dagaa* likely reflect differences in salting method and processing conditions. The reported salt concentrations (1.77 g/L brine versus 143 g/kg fresh fish) are expressed on different bases and therefore cannot be directly compared; thus, the differences in microbial counts cannot be attributed solely to the nominal salt concentrations but rather reflect the combined effects of the different salting mechanisms, boiling treatment, salt penetration, and subsequent dehydration (Birie et al. 2025). Nevertheless, salt penetration, boiling, and subsequent drying can collectively reduce microbial survival more by lowering water activity and inhibiting microbial growth (Basak et al. 2023; Tao et al. 2025).

The high yeast and mold counts in the fresh fish suggest unsanitary handling and storage conditions (Majumdar et al. 2024). These findings demonstrate that simple, low‐technology preservation practices, such as salting, preboiling, and drying, can substantially enhance microbial safety in small‐scale fish processing systems. Similar insights were also reported by Birie et al. (2025) and Maturin and Peeler (2001), who further observed that market‐sourced samples had consistently higher TPC, coliform levels, and aflatoxin contamination than landing‐site samples, largely due to adulteration during packaging, transport, and storage, as well as insect infestation and elevated moisture content. Cooking before consumption reduces consumer risk from these organisms; therefore, increasing counts are better interpreted as indicators of freshness decline and shelf‐life rather than direct consumer pathogen risk.

#### 4.1.2 Moisture Content

The dry‐salted, unboiled dried *Dagaa* showed higher moisture than boiled brine‐salted treatments, all exceeding the 12% safety limit (EAS 2014). Higher residual moisture reflects customary practices to increase saleable mass and reduce brittleness but compromises stability by enhancing microbial growth, lipid oxidation, and rancidity, thus shortening shelf life (Abdulmajid 2015; Kigozi et al. 2020; Kubiriza et al. 2021; Whiston et al. 2022). Effective drying requires strengthened process control, targeted capacity building, and the adoption of reliable moisture‐measurement instruments.

Boiling in a brine solution markedly enhanced drying kinetics, reducing the time to reach target moisture content from 20 to 5 h (a 75% increase in throughput). This improvement increases processing efficiency, an important performance determinant in women‐led fish processing units, while substantially limiting exposure to flies, dust, and other contaminants (Omagor et al. 2020; Sroy et al. 2023). The shortened drying period also reduces handling requirements and is particularly advantageous during the rainy season, when constrained sun‐drying windows heighten the risk of quality deterioration and postharvest losses.

#### 4.1.3 Proximate Composition

Boiling in a brine solution inactivates endogenous enzymes and microorganisms, facilitates water expulsion during drying, and reduces losses of protein through degradation, resulting in a higher apparent protein concentration in the final product as compared to dry‐salted, unboiled dried *Dagaa* (Abraha et al. 2018). Boiling in a salt solution for 12 min was identified as the optimum processing time, providing adequate freshness and safety assurance. Extending the boiling duration to 15 min did not confer additional freshness and safety benefits but resulted in substantial nutrient losses and the onset of undesirable physical changes, including deterioration in texture and morphological integrity. Prolonged boiling denatures muscle proteins, causing unfolding, aggregation, and leaching of fragments into the cooking medium, which reduces recoverable protein after drying (Yu et al. 2022). Similar losses with boiling time are reported in seafood and other muscle foods (Aberoumand et al. 2024). Precise control of boiling time, temperature, and brine concentration is critical for minimizing protein losses and preserving the nutritional quality of dried *Dagaa*.

The higher measured fat and crude fiber contents in *Dagaa* subjected to brine boiling followed by drying, compared to dry‐salted but unboiled dried treatments, were primarily due to differential moisture loss. Boiling in brine accelerates dehydration and muscle matrix restructuring, concentrating residual lipids and indigestible components (Fitri et al. 2022). This moisture‐concentration effect elevates crude fat and fiber on a dry‐weight basis, reflecting heat‐induced denaturation and water loss rather than absolute increases (Birie et al. 2025).

Previous studies have demonstrated that drying conditions can substantially influence the nutritional quality of *Dagaa* (Omagor et al. 2020). Drying on raised platforms enhances air circulation and promotes more uniform moisture removal, thereby reducing heat stress, microbial growth, and contamination that may contribute to protein degradation (Wu et al. 2024). In contrast, ground drying is often associated with greater moisture reabsorption and increased exposure to contaminants, which can accelerate nutrient losses (Omagor et al. 2020). Although the present study did not consider this factor, raised platform versus ground drying, variations in these practices may influence protein retention (Mgawe 2008).

#### 4.1.4 Micronutrients

Boiled, brine‐salted, and dried *Dagaa* exhibited higher Fe, Zn, Ca, and Mg concentrations than dry salting and drying alone, reflecting moisture loss, mineral uptake from the brine, and reduced leaching during dehydration, which collectively increases the apparent mineral content on a weight basis (Basak et al. 2023). Preboiling in a brine solution enhanced the retention and concentration of micronutrients in dried *Dagaa*, indicating that this processing step may improve the nutritional quality of the final product and should therefore be considered a recommended practice.

Dried *Dagaa* are commonly marketed in plastic liners without nutritional labelling, standardized serving sizes, or consumer advisories, including guidance on moderated consumption despite their high sodium content (Bavinck et al. 2023). Improving labelling and consumer information is therefore necessary to enhance consumer protection while supporting informed consumption and reducing avoidable nutrient and food waste (Kawarazuka and Béné 2011).

### 4.2 Physical Quality Changes

Boiling in a salt solution and sun‐drying reduced *Dagaa* length and width, with curvature, tissue detachment, and color darkening. These morphological changes reflect moisture loss, protein denaturation, structural degradation, and a consequent measurable mass reduction (Fitri et al. 2022). Reductions in edible mass translate to diminished nutrient retention (Ogonda et al. 2014). In particular, the detachment of the head is nutritionally important, given that fish heads are known to contain notable proportions of long‐chain omega‐3 polyunsaturated fatty acids (PUFAs), including EPA and DHA (Byrd et al. 2021; Bavinck et al. 2023). Furthermore, maintaining product integrity requires replacing saline water after each boiling cycle, but processors often skip this step and simply add more salt and water to save time (Diei‐Ouadi and Mgawe 2011; Ngwenyama et al. 2026).

Bulk density decreased after drying, with dry‐salted, unboiled, sun‐dried *Dagaa* showing lower bulk density than salted‐boiled sun‐dried *Dagaa* due to processing‐induced structural changes. Drying unboiled salted fish promotes gradual moisture loss, salt‐enhanced shrinkage, increased porosity, and looser particle packing, whereas boiling causes protein denaturation and fiber collapse, producing denser particles (Rahman and Driscoll 1994). Lower bulk density increases transport and storage costs per unit weight and may reduce trader margins where pricing is volume‐based, although it can enhance rehydration capacity and consumer appeal. In contrast, the higher bulk density of salted, boiled‐and‐dried *Dagaa* improves packaging efficiency and long‐distance market competitiveness (Ojijo et al. 2024). These observations suggest that processing choices influence not only product quality but also downstream logistics and market competitiveness and should therefore be considered in postharvest loss reduction and value addition strategies.

Prolonged boiling of *Dagaa* (15 min) in a brine solution increased structural softening and fragility, resulting in greater fragmentation and higher proportions of meal losses during the winnowing process prior to packaging. Salted, unboiled, sun‐dried *Dagaa* exhibited lower losses (~1%) compared with boiled treatments. Boiling in a salt solution increased fragmentation and material loss during winnowing, likely due to process‐induced physical damage, including head and tail detachment and body breakage. The resulting fragments/meal are usually sold for animal feed at around USD 0.39 per kg, compared with USD 3.33 for whole dried *Dagaa*, representing a substantial economic loss. The use of appropriately sized sieves instead of winnowing to improve processing throughput efficiency is recommended.

Salted, unboiled, sun‐dried *Dagaa* retained intact morphology but exhibited marked adhesion to drying surfaces and between fish, causing product losses. This stickiness is likely caused by exuded soluble proteins and lipids forming tacky films during slow dehydration, compounded by the hygroscopic properties of salt's enhancing surface moisture retention (Owaga et al. 2009; Birie et al. 2025). In the absence of blanching or boiling, native muscle proteins remain largely uncoagulated and undergo gradual denaturation during drying, which favors surface bonding rather than the rapid protein coagulation associated with heat treatment (Patterson et al. 2018; Tao et al. 2025). Similar adhesion phenomena during the drying of small fish and muscle foods have been attributed to protein denaturation, lipid migration, and salt‐moisture interactions at contact surfaces (Bellagha et al. 2007).

### 4.3 Boiling Equipment Energy Utilization Efficiency

The estimated annual firewood requirement for *Dagaa* processing in Kiyibuni landing site was about 42.5–50 t. Approximately 923–1086 fuelwood trees are required to produce this dry firewood, assuming typical smallholder yields of 46 kg per tree (Kumar et al. 2009; Muhamad et al. 2024). This huge firewood requirement has remarkable negative environmental consequences in the long run, including deforestation and increased carbon emissions from smoke (Fujimoto 2018; Wolff et al. 2021). Reducing such emissions is therefore critical, as it directly advances SDG 13 (climate action) and supports SDGs 7 (affordable and clean energy), 11 (sustainable cities and communities), and 12 (responsible consumption and production), while indirectly improving health, ecosystems, and sustainable economic outcomes.

This practice is further worsened by the common use of mangrove wood, which threatens vital marine ecosystems (Hamad et al. 2014; Dayalatha and Ali 2018; Carrasquilla‐Henao et al. 2019). Therefore, it is crucial to prioritize optimizing boiling times and exploring alternatives to traditional firewood boiling methods (Bille and Shemkai 2006).

### 4.4 Call for Action, Recommendations for Further Studies, and Limitations

There is a need to promote the adoption and scaling up of renewable‐energy‐based *Dagaa* boilers and dryers with simple, reliable control of key process parameters such as time, temperature, airflow, and humidity in the processing system (Ward and Peñarubia 2025). The use of efficient, climate‐smart energy technologies can reduce postharvest losses, improve product quality and safety, and lower environmental footprints while enhancing processor profitability. Strengthening technology transfer through closer collaboration among researchers, industry, equipment manufacturers, extension services, and policymakers is essential to accelerate the uptake, local adaptation, and commercialization of appropriate processing technologies (Odediran and Ojebiyi 2017).

Despite the availability of relevant technologies, climate adaptation is constrained by weak scaling rather than innovation deficits (Nyirenda et al. 2024). In Africa, inadequate investment, fragmented extension systems, poor technology transfer, and limited private‐sector engagement impede widespread adoption (Suuronen 2022; Abdul et al. 2024). Strengthening the innovation pipeline requires enabling policies, incentives for private actors, improved trade facilitation, gender responsiveness, and sustained human and physical capital investment (Odediran and Ojebiyi 2017; Obayopo and Alonge 2018; Nyirenda et al. 2024; Goulart et al. 2024).

Future research should evaluate the technoeconomic feasibility and life‐cycle environmental impacts of renewable‐energy boilers and dryers under real processing environments. Studies should incorporate a comprehensive assessment of microbiological quality and food safety to ensure product safety throughout processing, handling, and storage. In addition, research should focus on fine‐tuning and simplifying process control to avoid overprocessing *Dagaa*, thereby preserving nutritional quality, sensory attributes, and overall market value. Participatory trials with industry stakeholders are recommended to support technology refinement, capacity building, and scalable adoption across diverse fish value‐chain settings.

A limitation of this study is the absence of pH and titratable acidity measurements, and as well as texture analysis. Including these physicochemical indicators in future research would improve the understanding of salting effects on product stability, microbial inhibition, shelf life, and the mechanisms governing quality changes during processing and storage. Additionally, the salting treatments differed both in the method of salt application and in the basis and rate of salt application. One treatment used brine salting, whereas the other involved the direct application of salt to fresh fish. Consequently, the reported salt concentrations (1.77 g/L in brine and 143 g/kg fresh fish) are expressed on different bases and should not be considered equivalent salt doses. The treatments were selected to reflect the salting practices commonly used by processors at Kiyibuni and therefore provide an assessment under realistic processing conditions. However, because the amount of salt applied and the mode of application were not standardized across treatments, the observed differences cannot be attributed exclusively to the salting method or boiling. The independent effect of boiling cannot be determined from the present experimental design. Future studies should control salt application on a common basis, preferably by standardizing the salt‐to‐fish ratio and/or achieving comparable salt concentrations in the fish tissue, to enable a more direct comparison of salting methods.

## 5 Conclusion

This study demonstrates that boiling, salting, and drying methods influence the nutritional quality, drying behavior, physical quality attributes, and energy performance of *Dagaa* processed under smallholder fishery conditions. Overall, the findings support the adoption of a 12‐min salt‐boiling treatment before drying as a technically optimal processing approach that balances extended shelf life with consumer acceptability and profitability. Short‐duration steam blanching may represent a promising alternative, although it was not evaluated in the present study.

At the policy level, targeted investment in climate‐smart, energy‐efficient *Dagaa* processing technologies is essential to reduce dependence on firewood, thereby mitigating pressure on local forest resources while enhancing the resilience and sustainability of regional food supply systems. From an extension perspective, processing guidelines should discourage prolonged boiling, as a boiling duration of approximately 12 min provides an optimal balance between nutrient retention, product safety, quality, and drying efficiency. Extended boiling to 15 min did not confer additional microbiological safety benefits; however, it resulted in significant reductions in protein, zinc, calcium, and magnesium concentrations.

The combined application of boiling and salting prior to drying should be promoted, given its potential to shorten processing time, enhance product quality and safety, and reduce nutritional losses. A key challenge during processing is minimizing mineral losses associated with leaching into the boiling medium. Improved postharvest technologies can reduce these losses and enhance quality; however, they may generate nutrition trade‐offs. Enhanced shelf life and product value may stimulate export demand, potentially reducing domestic availability and affordability of nutrient‐dense *Dagaa* for low‐income consumers. Seasonal and climate variability may further mediate these effects.

The adoption and scaling of improved *Dagaa* processing practices could enhance food quality and safety, reduce postharvest losses, increase income generation, and improve nutritional outcomes. More broadly, the implementation of these optimized processing practices would contribute to sustainable aquatic food systems and support progress toward achieving the United Nations Sustainable Development Goals (SDGs).

## Author Contributions

**Patrick Ngwenyama:** conceptualization, methodology, investigation, validation, writing – original draft, software, formal analysis, data curation, project administration, funding acquisition, visualization, resources. **Patrick Kitabi:** investigation, writing – review and editing, methodology. **Derrick Tunje:** investigation, data curation, writing – review and editing. **Emmanuel Masoud:** investigation, methodology, data curation, writing – review and editing. **Pamela Odhiambo:** investigation, methodology, writing – review and editing, data curation. **Aditya Parmar:** supervision, resources, project administration, formal analysis, investigation, conceptualization, methodology, validation, visualization, funding acquisition, writing – review and editing. **Patrick Kimani:** conceptualization, investigation, validation, writing – review and editing, supervision, funding acquisition. All authors have read and approved the final version of the manuscript. Corresponding Author had full access to all of the data in this study and takes complete responsibility for the integrity of the data and the accuracy of the data analysis.

## Funding

Financial support for this research was provided by the Foreign, Commonwealth & Development Office (“FCDO”), UK and ASIA‐AFRICA BLUETECH SUPERHIGHWAY PROJECT under the Climate and Ocean Adaptation and Sustainable Transition programme (COAST), FCDO Project Number: 301203.

## Ethics Statement

The study protocol was reviewed and approved by Jaramogi Oginga Odinga University of Science and Technology Committee in accordance with Kenyan laws, ethical standards, and national guidelines governing research activities. The study did not involve clinical procedures, the collection of biological specimens from human participants, or the acquisition of personal identifying information. Research activities were limited to non‐sensitive field observations, stakeholder interviews, and the collection of dried *Dagaa* samples for laboratory analysis.

All interviews were conducted with the informed consent of participants, and the confidentiality and anonymity of respondents were maintained throughout the study. The study was therefore considered to present minimal risk to participants and was conducted in accordance with established ethical principles for research involving human participation.

## Consent

The study obtained informed consent from all participants. Participants were informed about the study objectives and procedures, and they were assured that no personal information such as names, contact details, or identifying characteristics would be collected or recorded. Participation was entirely voluntary.

## Conflicts of Interest

The authors declare no conflicts of interest.

## Data Availability

The datasets used and analyzed during the current study are available from the corresponding author on reasonable request. The data are not publicly available due to ethical restrictions.
